# Population-based study and a scoping review for the epidemiology and seasonality in and effect of weather on Bell’s palsy

**DOI:** 10.1038/s41598-021-96422-4

**Published:** 2021-08-20

**Authors:** Min Hee Kim, So Young Park

**Affiliations:** Department of Ophthalmology, Otolaryngology and Dermatology of Korean Medicine, Kyung Hee University Hospital at Gangdong, Dongnam-ro 892, Seoul, 05278 Republic of Korea

**Keywords:** Epidemiology, Neurological disorders

## Abstract

The association between weather-related variables or seasons and the development of Bell’s palsy (BP) is controversial. This study aimed to evaluate the incidence and clinical characteristics of BP and assess the effects of meteorological factors on seasonal and monthly incidence. This retrospective population-based study used data from the Korean Health Insurance claims database (NHICD) from 2010 to 2018, in which annual, seasonal, and monthly incidence rates and age and sex distributions were calculated. A multivariate linear regression and autoregressive integrated moving average (ARIMA) model was used to determine the association between the seasonal and monthly incidence of BP and meteorological factors, including average wind speed, temperature, relative humidity, and atmospheric pressure. We also conducted a scoping review of the literature on epidemiological and seasonality studies of BP in the past 30 years and summarized them in a table for easy comparison with other studies. In this study, the incidence rate of BP increased over 9 years (from 12.86 to 19.92 per 100,000 persons) and was the highest in patients in their 60s (31.6/100,000 persons). The seasonal incidence of BP was the highest in autumn and showed a significant difference compared with spring (coefficient − 0.318, *p* = 0.003) and summer (coefficient − 0.463, *p* < 0.001), adjusting the year. In the ARIMA analysis, the autocorrelation of the monthly and seasonal lag in the raw data disappeared after adjustment of the seasonal (or monthly) and longitudinal changes, indicating no additional trends outside the seasonal (or monthly) longitudinal changes. The seasonal and monthly incidence of BP was related to low temperature (*p* = 0.002), high atmospheric pressure (*p* = 0.034), and low relative humidity (*p* < 0.001) in the multivariate linear regression. In contrast, in the ARIMA analysis, after adjusting for seasonality, month, and trends, there were no significant meteorological factors associated with the monthly or seasonal incidence rate. In the past 30 years, 12 studies have reported on the prevalence or incidence of BP, and 14 have reported on the relationship between seasons, weather, and incidence. These results indicate that BP is more common among the elderly, and the incidence of BP is increasing due to an aging society, increased medical accessibility, and lifestyle changes. The data also indicate that the onset of BP is associated with low temperature and humidity; however, in the climate zone with extreme temperature and humidity differences between the coldest and hottest months, it is assumed that the marked decrease in temperature (autumn) has more influence on the outbreak of BP than does the actual cold temperature (winter).

## Introduction

Bell’s palsy (BP) is a rapid unilateral facial nerve paresis (weakness) or paralysis (complete loss of movement) of unknown cause^1^. BP affects individuals across various age groups and in both sexes, with an annual incidence ranging from 13 to 107 per 100,000 persons^2–10^.

Although it has been suggested that anatomical defects, viral infection, ischemia, inflammation, and cold exposure are associated with the onset of BP, the exact pathophysiology of BP remains unclear^11^. Previous studies have demonstrated that weather-related variables, namely, temperature, atmospheric pressure, wind speed, humidity, and seasonality, are associated with BP development^3,12–24^; however, because of the selection and observation bias due to the design of those studies, the study results remain controversial. Therefore, to identify the exact relationship between weather and BP, a seasonality study with a larger sample size and long-term observation is required in countries with four distinct seasons.

The epidemiology of BP varies according to the methodological differences and populations surveyed. In epidemiological studies, a population-based design reduces sampling bias and is capable of accurately assessing the characteristics of relatively rare disorders. In a worldwide literature review, only one population-based study was conducted in the UK in 2002, which reported the yearly incidence rate^4^. In Asian countries, no studies have reported the incidence rate in the last 30 years, and only one Korean study evaluated the prevalence of sequelae of facial palsy^25^.

To the best of our knowledge, no other study has reported the incidence of BP using a population-based design in an Asian country or seasonal variation in BP using a population-based design anywhere in the world. Therefore, the aims of this study were to determine the annual, seasonal, and monthly incidences and clinical characteristics of BP and to assess the effects of wind speed, temperature, atmospheric pressure, and humidity on BP development. The seasonal and monthly incidences of BP were based on the 9-year data from the Korean National Health Insurance Claims Data (NHICD) and a scoping review of epidemiological and seasonality BP studies was conducted.

## Result

### Incidence

**Figure 1:**
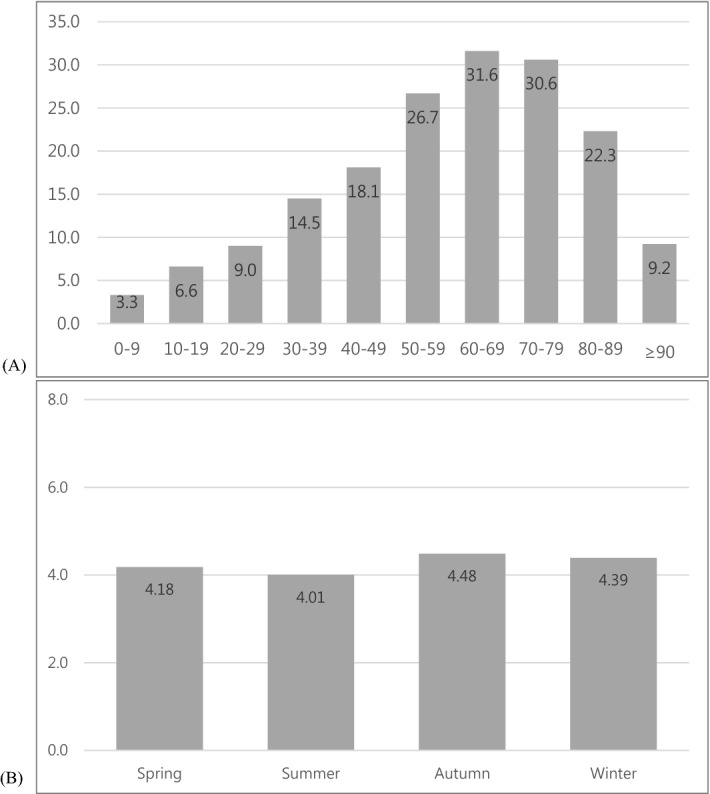
Incidence of Bell's palsy by age group (**A**) and seasons (**B**) (2010–2018, per 100,000 persons, descriptive statistics).

The overall incidence of BP in men and women has increased over the last 9 years (from 12.86 to 19.92 per 100,000 persons, Table 1), with no significant difference between the sexes. The incidence of BP increased with age, with the highest rates in the 60-year-olds, and it decreased in the 70-year-olds [31.6 in 60s, 30.6% in 70s, and 22.3 in 80s (per 100,000 persons), Fig. 1]. This trend was the same between 2010 and 2018.

**Table 1:** Incidence of Bell's palsy by sex.

Year	Overall		Men		Women	
	No	Rate	No	Rate	No	Rate
2010	6416	12.86	3157	12.64	3259	13.09
2011	7450	14.87	3713	14.80	3737	14.93
2012	8361	16.61	4144	16.45	4217	16.76
2013	8171	16.16	4119	16.29	4052	16.03
2014	8886	17.50	4485	17.68	4401	17.33
2015	9057	17.78	4649	18.26	4408	17.29
2016	9477	18.54	4772	18.69	4705	18.39
2017	9880	19.29	4942	19.32	4938	19.25
2018	10,219	19.92	5230	20.43	4989	19.41
Total	77,917		39,211		38,706

### Seasonal variation

In the multivariate linear regression, there was a significant difference between the incidence of BP in autumn and summer (coefficient − 0.463, *p* < 0.001) and autumn vs. spring (coefficient − 0.318, *p* = 0.003), after adjusting for the year. However, the difference was not significant among the other seasons (Fig. 1 and Table 2). In the ARIMA analysis, autocorrelation for the monthly and seasonal lag in the raw data disappeared after adjustment of the seasonal (or monthly) and longitudinal changes (monthly; *p* < 0.001 to *p* = 0.775, seasonal; *p* < 0.001 to *p* = 0.732). This result indicates that there are no additional trends outside the seasonal (or monthly) longitudinal changes; in other words, the presence of seasonal (or monthly) and longitudinal changes.

**Table 2:** The seasonality and the association between the seasonal and monthly incidence of Bell’s palsy and meteorological factors.

Variable	Coefficient	*p* value	95% confidential interval
**Seasonality**			
Autumn	1	1	1
Spring	− 0.318	0.003*	− 0.520 to 0.116
Summer	− 0.463	0.000**	− 0.665 to 0.260
Winter	− 0.116	0.252	− 0.318 to 0.086
**Meteorological factors (adjusted for a year)**			
*Seasonal incidence*			
Wind speed	− 0.120	0.515	− 0.477 to 0.237
Temperature	− 0.014	0.002*	− 0.022 to − 0.001
Atmospheric pressure	0.029	0.034*	− 0.027 to − 0.002
Relative humidity	− 0.014	0.000**	0.019 to 0.038
*Monthly incidence*			
Wind speed	− 0.014	0.722	− 0.088 to 0.061
Temperature	− 0.004	0.000**	− 0.006 to − 0.002
Atmospheric pressure	0.007	0.000**	0.005 to 0.010
Relative humidity	− 0.003	0.023*	− 0.006 to − 0.001

Multivariate linear regression analysis.

*p < 0.05 **p < 0.001.

### Meteorological factors

**Figure 2:**
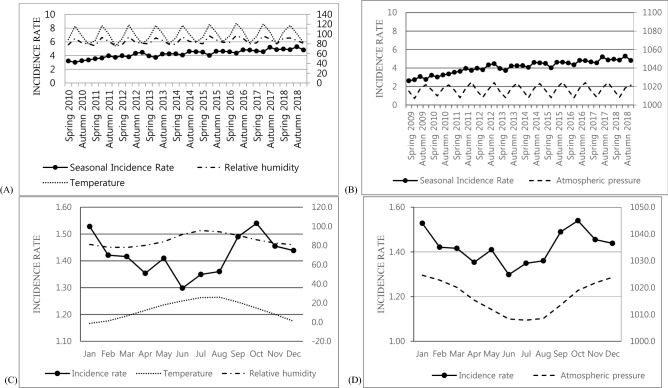
Seasonal (**A**, **B**) and monthly (**C**, **D**) incidence of Bell's palsy [(**A**, **C**) average temperature and average relative humidity, (**B**, **D**) average atmospheric pressure, per 100,000 persons].

In the multivariate analysis, there was a significant negative correlation between average temperature (coefficient − 0.014, *p* = 0.002), average relative humidity (coefficient − 0.014, *p* < 0.001), and seasonal incidence and a significant positive correlation with average atmospheric pressure (coefficient 0.029, *p* = 0.034); however, there was no significant difference in average wind speed (coefficient − 0.120, *p* = 0.515; Table 2 and Fig. 2). This correlation was also the same in the multivariate analysis for meteorological factors and monthly incidence (Table 2 and Fig. 2). On the other hand, in the ARIMA analysis, after adjusting for seasonality, month, and trends, there were no significant meteorological factors associated with the monthly or seasonal incidence rate (Table 3).

**Table 3:** ARIMA analysis for meteorological factors.

Independent variable	β	SE	t-value	*p* value
**Seasonal (Reference group: Winter)**				
Intercept	2.358	15.515	0.152	0.880
Wind speed	0.008	0.087	0.089	0.930
Temperature	0.001	0.012	0.119	0.907
Atmospheric pressure	− 0.003	0.015	− 0.169	0.867
Relative humidity	0.003	0.004	0.777	0.445
**Monthly (Reference group: December)**				
Intercept	1.997	5.105	0.391	0.696
Wind speed	0.059	0.043	1.372	0.173
Temperature	0.006	0.005	1.020	0.310
Atmospheric pressure	− 0.002	0.005	− 0.423	0.673
Relative humidity	0.000	0.001	0.113	0.910

### Reported Bell’s palsy incidence and seasonality

In the past three decades, 12 studies have reported on the prevalence or incidence of BP, and 14 have reported on the relationship between seasons, weather, and incidence (Table 3). Annual incidences were reported from other countries such as the USA^5,26^, Canada^6^, United Kingdom^4^, Spain^3^, Denmark^7^, Italy^2,8^, and Egypt^9,10^, but no Asian studies have reported the incidence. There have also been no studies that used a population-based design based on the entire population of their respective countries.

## Discussion

This study had the following findings: (1) the incidence rate of BP in Korea showed a rapid yearly increase from 2010 to 2018; (2) BP was the most common among those in their 60s; (3) the seasonal incidence of BP was highest in autumn, followed by winter, spring, and summer; and (4) seasonal and monthly incidences of BP were related to low temperature, low humidity, and high atmospheric pressure.

In the present study, the annual incidence rate per 100,000 persons was 12.86% in 2010, which increased to 19.92 in 2018. In the past 30 years, there have been many reports on the prevalence and incidence of BP (Table 4). The reported annual incidence of BP based on regional medical services ranged from 13.1% to 42.8 per 100,000 persons^5–7,26^, and results from hospital-based studies ranged from 24.1 to 53.3% per 100,000 persons^3,8^. The only epidemiological study of BP in Asian countries, which was based on regional medical services and was published in 1988, reported that the incidence of BP was 30 per 100,000 persons in a prefecture of Japan^27^. The only population-based study according to national medical services was conducted in the UK from 1992 to 1996, with a reported incidence rate of 20.2/100,000 persons^4^. In that study, BP was defined based on the diagnosis code, but the prescription of steroids, which was included in our study, was not considered. This wide range of incidence rates by countries may be due to differences across studies with regard to inclusion criteria, such as diagnostic codes, tests, prescriptions, subjects, regions, and methodologies. In addition, the proportion of the population for those aged 65 years and over was 15.9% in the UK^28^, while the proportion of this same population increased from 11.3% in 2010 to 14.3% in 2018 in South Korea^29^. Therefore, it should be noted that the increasing incidence of BP may be because the number of patients is actually increasing, based on an aging society.

**Table 4:** Reported Bell's palsy incidence and seasonality in studies conducted in the past three decades.

	Year	Location	Study period	Data collection method	Criteria	Size of population	Prevalence/Incidence (per 100,000 persons)	Sex ratio (F/M)
**Incidence**								
Brandenburg and Annegers^26^	1993	USA	1974–1982	Regional medical database	BP diagnosed	91,449	–/22.8–32.7 (according to sex and region)	1.5
Savettieri et al.^2^	1996	Italy	1984–1987	Door-to-door survey	BP diagnosed	11,901	642.8/52.8	1.0
Myers et al.^3^	1999	Spain	1980–1996	Single hospital-based data	BP diagnosed	465,000	–/24.1	1.2
Morris et al.^6^	2002	Canada	1997/8/1–11/15	Regional medical database	BP diagnosed + MRI (for volunteers)			1.2
		Grater Toronto area				4,987,008	–/15.2	–
		nova scotia				936,587	–/13.1	–
Peitersen^7^	2002	Denmark	25 years	Regional medical database	BP diagnosed	2570	–/32.0	1.1
Campbell and Brundage^5^	2002	USA	1997–1999	Regional medical database	BP diagnosed	2,761,496	–/42.77	0.2
Rowlands et al.^4^	2002	UK	1992–1996	National medical database	BP diagnosed	35,000,000	–/20.2	1.0
Nicoletti et al.^30^	2002	Bolivia	1994–1996	Door-to-door survey	BP diagnosed	10,124	1110/–	1.0
Monini et al.^8^	2010	Italy	2006–2007	Multicenter hospital-based data (4)	BP diagnosed + CT, MRI	714,509	–/53.3	0.9
El Tallawy et al.^9^	2013	Egypt	2006–2008	Door-to-door survey	BP diagnosed	62,583	192.18/51.89	1.6
Khedr et al.^10^	2016	Egypt	2013–2015	Door-to-door survey	BP diagnosed	9303	161/107	1.0
Chang et al.^25^	2016	Korea	2010–2012	Door-to-door survey	H-B grade 3 or higher	23,533	120/–	1.2

	Year	Location	Study period	Data collection method	Criteria	Size of population	Season/month	Weather (No significance)
**Seasonality**								
Myers et al.^3^	1999	Spain	1980–1996	Single hospital-based data	BP diagnosed	1906	Summer↓	
Danielides et al.^12^	2001	Greece	1995–1999	Single hospital-based data	BP diagnosed + PTA, lab., electrophysiologic test	171		- (Temp, Atm, Hum)
de Diego et al.^13^	2002	Spain	1992–1996	Single hospital-based data	BP diagnosed	662		Low temperatures (Atm, Air pollutants)
Spengos et al.^14^	2006	Greece	1990–2004	Single hospital-based data	BP diagnosed	1,252	Summer↓ Autumn ↑	
Narci and Uğur^15^	2012	Turkey	2007–2010	Single hospital-based data	BP diagnosed	533	Spring↓ Winter↑	
Hsieh et al.^16^	2013	Taiwan	3 years	Single hospital-based data	BP diagnosed	775	Dec–Feb ↑ (only in men)	
Jeon et al.^17^	2013	Korea	2007–2011	Single hospital-based data	BP diagnosed	488	No significance	- (Temp, Atm, Hum, Wind speed)
Jamil et al.^18^	2013	Saudi Arabia	2011–2012	Single hospital-based data	BP diagnosed + PTA, lab., electrophysiologic test	403	Autumn↑	
Kokotis and Katsavos^19^	2015	Greece	1993–1999	Single hospital-based data	BP diagnosed	561	Oct–Apr↑	Low temperature High WCF^a^ (HCTF, HCTR, TFR, TRR, Wind speed)
Yang et al.^20^	2016	China	2007–2008	Multicenter hospital-based data (6)	BP diagnosed	1310	Winter↑	
Zhao et al.^21^	2017	China	2012–2014	Single hospital-based data	BP diagnosed	372	Summer↑	
Erdur et al.^22^	2018	Germany	2010–2017	Single hospital-based data	BP diagnosed	46,289	Dec↑ July↓	
Franzke et al.^23^	2018	Germany	2006–2016	Multicenter hospital-based data (3)	BP diagnosed	825		High atmospheric pressure (Temp, Hum)
Yilmaz et al.^24^	2019	Turkey	2005–2016	Single hospital-based data	BP diagnosed	816	Winter↑	

*BP* Bell’s palsy, *PTA* pure tone audiometry, *Temp* temperature, *Atm* atmospheric pressure, *Hum* humidity, *WCF* wind chill factor, *HCTF* highest continuous temperature fall, *HCTR* highest continuous temperature rise, *TFR* temperature fall rate, *TRR* temperature rise rate.

^a^A measure meant to represent the hypothetical air temperature that would, under the conditions of no wind, lead to the same heat loss from unclothed human skin, as the actual combination of air temperature and wind speed does.

In this study, BP showed the highest incidence rate among patients in their 60s, 70s, and 50s (in descending order). The age distribution of BP has been reported in several studies. Some studies have reported that BP has the highest incidence rate among those in their 20s to 50s^5,7,9,10,21^ while others have reported that the incidence of BP is most common among those over 60 years^2,4,25,26,30^. In a UK study based on national medical services, the incidence rate of BP peaked in the population aged 75 years and above^4^, which is consistent with our results. The rapid progression to an aging society, increase in medical accessibility, and lifestyle changes could be the reasons for the increase in the incidence rate due to aging in our study and the UK study^4^. Results from these studies suggest that other developed countries with high medical accessibility and an aging population might have a similar age distribution of BP and the highest incidence rate in the elderly.

In this study, the seasonal incidence of BP was the highest in autumn and showed significant differences between spring and summer in the multivariate analysis. Many studies in various countries and climates have reported on the seasonal variability of BP based on over 3 years of observation (Table 4); studies conducted in Turkey^15,24^ Taiwan^16^, Greece^19^, and Germany^22^, which are countries in the oceanic or Mediterranean climate zones, have reported that the seasonal incidence of BP is significantly higher in winter. In comparison, the Korean climate features a humid continental climate, called "Dwa" in the Köppen climate classification^31^. This climate zone has four distinct seasons, with a very hot and humid weather during the summer season and a very cold and dry weather during the winter season (− 1.3 °C and 81.1% in January and 26.0 °C and 94.3% in August; the monthly average from 2009 to 2018). Therefore, spring and autumn are shorter, and the temperature change is more rapid in Korea than in the countries of previous studies.

Several studies have also reported relationships between meteorological factors and the incidence of BP (Table 4). Low temperatures^13,19^, high wind chill factors^19^, and acute changes in atmospheric pressures^23^ were reported to be significantly associated with the risk of BP. Humidity^12,17,23^, wind speed^17^, and air pollutants^13^ were also analyzed in previous studies; however, no significance has been reported. Consistent with these previous reports, we found that the seasonal and monthly incidence of BP was related to low temperature, low humidity, and high atmospheric pressure. Combining previous studies and our current study, the onset of BP is associated with low temperature and humidity; however, in the climate zone with extreme temperature and humidity differences between the coldest and hottest months, it is supposed that the marked decrease in temperature (autumn) has a greater influence on the outbreak of BP than the actual cold temperature (winter). As described above, the effects of seasons and meteorological factors differ by climate and methodology for data collection and statistics, reinforcing the need for other well-designed studies from other climate zones.

Our study had several implications. This is the first study to observe the annual incidence rate and age distribution of BP in an Asian country and the first to analyze the relationship among seasons, changes in weather, and the risk of BP worldwide, based on national population-based data, and verified BP diagnosis. As the Health Insurance Research and Assessment (HIRA) data included nearly the entire population, it was appropriate to use them to represent the South Korean population with enough strength to reduce any sampling bias. We also conducted a scoping review of epidemiological and seasonality studies for BP in the past 30 years and summarized them in one table to make it easier to understand the differences between the published works.

Despite these findings, this study has some limitations. In the analysis of the meteorological factors, we used both multivariate linear regression and the ARIMA model, and the statistical significance was found only in the former analysis. Although the effect of time was adjusted in the linear regression, the interpretation of our results may be controversial, because the ARIMA models are recognized as having more statistical power in the analysis of the effect of meteorological factors. In addition, we used the average monthly weather of the country and the average incidence rate in the analysis of the relationships between weather and incidence rate, instead of using geographically close weather, and the weather on the day of onset for each patient. This limitation may decrease the sensitivity of the analysis and may have influenced the non-statistical significance of the ARIMA analysis. From this study, the recent epidemiological trend of BP was clearly identified with large population-based data; however, in order to confirm the effect of weather, further study based on daily, geographically close weather data would be desirable.

## Conclusions

In conclusion, we conducted a national population-based retrospective cohort study that observed 9-year data in Korea to determine the annual, seasonal, and monthly incidence and clinical characteristics of BP and assess the effects of wind speed, temperature, atmospheric pressure, and humidity on the seasonal and monthly incidence of BP. We also conducted a scoping review of the epidemiological and seasonal BP studies. These results indicate that BP is more common among the elderly, and the incidence of BP is increasing due to an aging society, increased medical accessibility, and lifestyle changes. The data also indicate that the onset of BP is associated with low temperature and humidity; however, in the climate zone with extreme temperature and humidity differences between the coldest and hottest months, it is assumed that the marked decrease in temperature (autumn) has more influence on the outbreak of BP than does the actual cold temperature (winter). In this study, we clearly identified the recent epidemiological trend of BP using a large population-based data set; however, in order to confirm the effect of weather, further study based on daily, geographically close weather data would be desirable.

## Methods

### Study design and data source

This study used data from the NHICD provided by the HIRA service of Korea from January 2010 to December 2018. In Korea, 97% of the population are covered by national health insurance, and the remaining 3% of the population are covered by the medical aid program^32^. The NHICD contains information on all patients, including diagnostic codes, treatment procedures and details of prescriptions, expenditure amounts, and personal information in outpatient and inpatient care, making it a good resource for identifying disease prevalence and medical behavior^33^. In the NHICD, the Korean Classification of Diseases, seventh revision (KCD-7), a system similar to the International Classification of Disease (ICD-10), is employed as a system of diagnostic practice codes. All methods were performed in accordance with the relevant guidelines and were approved by the Institutional Review Board (IRB) of the Kyung Hee University Hospital at Gangdong (IRB No.2020-03-008). Informed consent was unnecessary because this study involved minimal risk to human subjects, and its requirement was waived by the IRB of the Kyung Hee University Hospital at Gangdong (IRB No.2020-03-008).

### Study population

To increase the diagnostic accuracy of BP, we only enrolled patients whose records showed a prescription for BP medicine as well as an appropriate KCD-7 diagnostic code, which diagnosed BP as G510. Therefore, we only included patients who were diagnosed with BP more than twice and received a prescription of prednisolone (193302ATB) or methylprednisolone (217001ATB), as these are the most commonly used first-line medical treatment for BP^1^. Prednisolone is typically prescribed in a 10-day tapering course^1^, and most patients with BP are prescribed a 5-day maximum dose of prednisolone as the first treatment and a 5-day tapering dose of prednisolone as the subsequent treatment. In rare cases, clinicians unconsciously copy the diagnosis from the previous treatment at the next visit to treat other diseases, as prednisolone is often prescribed for many inflammatory diseases. Therefore, if we had included patients who had only been diagnosed once, it would be possible to include patients prescribed steroids for other inflammatory diseases. For this reason, we decided to only include patients who received a prescription of prednisolone for their BP (G510) more than twice^34^. To identify newly diagnosed patients, we defined the washout period as 2-years, and incident cases were defined as patients diagnosed with BP for the first time in the year.

### Incidence, seasonal variation, and meteorological factors

The incidence rates of BP between 2010 and 2018 were calculated. The incidence of BP was calculated using the number of incident cases divided by the mid-year population provided by the Korean Statistical Information Service^29^. The total monthly incidence of BP and the incidence of BP according to the patients’ sex, age, and month of diagnosis were investigated. To examine seasonal variations in the incidence of BP, March to May was classified as “spring,” June to August as “summer,” September to November as “autumn,” and December to February as “winter.” In addition, we calculated the seasonal average temperature, relative humidity, atmospheric pressure, and wind speed using data reported by the Korea Meteorological Administration^1^.

### Scoping review

The results of incidence and seasonality varied greatly according to the methodological differences, populations surveyed, and region in which the study was conducted. To observe the heterogeneity of incidence and seasonality of each study and to increase the quality of this epidemiological study, we reviewed the studies conducted over the past three decades. We searched PubMed, EMBASE, and Web Science Core Collection for studies published from 1990 to the present (September 24, 2020). The search terms used were (‘bell palsy’ OR ‘bell’s palsy’) AND (‘epidemiology’ OR ‘incidence’ OR ‘seasonal’ OR ‘seasonality’ OR ‘weather’ OR ‘meteorological’). We included studies that included self-generated epidemiological data and excluded studies that included patients with other diseases. Two researchers (MHK and SHK) carried out the study selection independently and discussed their differences and read all the selected articles. We extracted publication year, location, study period, data collection method, subject criteria, size of population, and study results (prevalence/incidence and sex ratio for incidence studies and relationship with season, month, or weather for seasonality studies).

### Statistical analysis

Descriptive statistics are presented as absolute and relative frequencies. Multivariate linear regression analysis adjusted for a year was performed to determine the seasonality and association between the seasonal incidence of BP and meteorological factors. We also adopted the ARIMA regression method to determine the incidence rate and calculated the autocorrelation coefficients and autocorrelation functions for all time lags. It is also used to evaluate the associations between meteorological factors and monthly and seasonal incidence rates after adjusting for the time-trend effect. All statistical analyses were performed using SAS EG 6.1 (SAS Institute, Cary, NC, USA) and R 3.5.1 (Foundation for Statistical Computing, Vienna, Austria).
